# Zika, Nipah and Kala-azar: Emerging lethal infectious diseases amid COVID-19 as an escalating public health threat in South India

**DOI:** 10.1016/j.amsu.2022.103972

**Published:** 2022-06-15

**Authors:** Utkarsha Uday, Lakshmi Jyothi Tadi, Zarmina Islam, Parvathy Mohanan, Shamas Ghazanfar, Maryam Salma Babar, Sumayya Ismail

**Affiliations:** a West Bengal University of Health Sciences, Kolkata, India; b Department of Microbiology, All India Institute of Medical Sciences, Bibinagar, India; c Faculty of Medicine, Dow Medical College, Dow University of Health Sciences, Karachi, Pakistan; d Faculty of Medicine, Medical University Sofia, Bulgaria; e Dubai Medical College for Girls, Dubai, United Arab Emirates

**Keywords:** Black fever, COVID-19, Delta mutant, Emerging infectious disease, Kala-azar, Nipah, Omicron, Syndemicity, Visceral leishmaniasis, Zika, BSL-3, biosafety level 3, COVID-19, Coronavirus disease-19, GDP, gross domestic product, ICU, intensive care unit, nCoV, novel Coronavirus, NiVD, Nipah virus disease, NRHM, national rural health mission, NVBDCP, national vector borne disease control programme, PKDL, post Kala-azar dermal leishmaniasis, PPE, personal protective equipments, SARS-CoV-2, severe acute respiratory syndrome-Coronavirus-2, SOP, standard operating procedures, VL, Visceral leishmaniasis, WHO, world health organization, ZVD, Zika virus disease

## Abstract

As of 6 June 2022, a sum 25,782 of active cases and 524,701 deaths due to Coronavirus disease-19 (COVID-19) have been recorded in India. Stewing in the flares of the pandemic, Kerala is entwined in the wrath of multiple emerging infectious diseases.

India, a home to 1.3 billion people, recently faced a devastating second wave of COVID-19 during May of 2021, with a ruckus of chronic shortage of medicine, oxygen supplies, ventilators, besides, being challenged by secondary infections and chronic health ailments. The state of Kerala, alone contributes to 50% COVID-19 caseload, besides, recent simultaneous outbreaks of Zika Virus Disease (ZVD), Nipah Virus Disease (NiVD) and Kala-azar (black fever) on July 8, September 5 and 8, 2021 respectively. Syndemicity and a high case fatality rates of these highly contagious diseases coupled with post infection sequelae, overwhelm the already fragile healthcare system. Thus, these lethal infectious diseases along with an anticipated third wave of COVID-19 pose a serious public health threat in and around South India. With this narrative review, we aim to discuss the challenges that the emergence of intersecting outbreaks of Zika, Nipah, Kala-azar presents with, in the nation, amidst the global pandemic of COVID-19 and provide recommendations so as to help alleviate the situation.

The syndemicity of COVID-19 with other infectious diseases, calls for adequate surveillance and monitoring of diseases’ outbreaks. To avoid the worst situations like pandemic, the health ministry, public and private health stakeholders in India should strengthen the public healthcare delivery system and providence of quick medical facilities to control the rate of mortality and morbidity during outbreaks.

## 1 Introduction

Infectious diseases continue to significantly contribute to human and animal morbidity as well as mortality. India, a home to 1.3 billion people, faced a devastating third wave of Coronavirus disease-19 (COVID-19 during January of 2022 [1]. The nation which is currently spending hardly 3% of its gross domestic product (GDP) on healthcare, faced a chronic shortage of medicine and oxygen supplies and space in intensive care units (ICU), and is challenged by secondary infections such as mucormycosis and chronic health ailments prevalent among the Indian population [2,3]. As of 6 June 2022, 43,181,335 cases have been recorded, with major contributions from the state of Maharashtra, seconded by Kerala [1]. Despite the vaccination against COVID-19 in India being rolled out on 16 January 2021, hardly 44% had been fully vaccinated within a year, which clearly shows that the vaccination policy is still inadequate and not undisputedly equitable [4,5]. As of 6 June 2022, close to 1.86 billion Indians have been vaccinated, comprising of 28,787,694, Keralites accounting for 81.12% of the total state population, with 101% being above 18 years of age and that of 83% under 15–18 years of age [6]. However, emerging breakthrough infections in the state, mutant delta and omicron variants are alarming [7,8].

Stewing in the flares of the pandemic, Kerala is entwined with the wrath of multiple emerging lethal infectious diseases. The state alone contributes to 50% of the COVID-19 caseload, besides, recent simultaneous outbreaks of ZVD, NiVD, and VL, also called kala-azar or black fever, on July 8, September 5, and 8, 2021 respectively [1,6,[9], [10], [11]]. The high case-fatality rates of these diseases coupled with post-infection sequelae overwhelm the already fragile healthcare system. Moreover, the possibility of a more devastating fourth wave is a cause for concern, given the surfeit of festive gatherings. These emerging infectious diseases pose a serious threat to public health in and around Kerala.

Syndemicity among COVID-19 and other infectious diseases:

‘Emerging infectious diseases’ are those whose incidence in humans have increased in the past two decades or threaten to increase in the near future [12]. The term ‘syndemic’ refers to two or more epidemics interacting synergistically and contributing to the clustering of excess disease burden in a population which is more than their sum. It is determined by the biological, social, environmental, and political factors prevalent in the respective populations [12]. Despite having varied aetiopathogenesis, these diseases may share a common spectrum of signs and symptoms. The severe acute respiratory syndrome-Coronavirus-2 (SARS-CoV-2) or novel Coronavirus (nCoV) causing COVID-19 spreads via droplets and fomites; Flavivirus causing ZVD is transmitted by the vector female *Aedes* mosquitoes; and the Paramyxovirus causes NiVD, whereas *Leishmania* protozoa is transmitted via the bite of infected female sandflies, resulting in visceral leishmaniasis (VL) [9,[13], [14], [15]]. Major symptoms including fever, low blood counts, hepatomegaly, chronic dry cough, and disorientation are similar among COVID-19, ZVD, NiVD, and VL, despite the incubation period varying from up to 14 days for these viral infections and up to 4 months for VL [9,[13], [14], [15]]. Moreover, individuals of geriatric age or immunocompromised individuals, pregnant women, children under the age of five years, healthcare or laboratory workers, animals, carcasses, or cadaver handlers encompass the risk groups shared among these diseases. Chronic ailments, travel, and overcrowding further amplify this risk [9,[13], [14], [15]].

## 2 Challenges

Multiple outbreaks of communicable diseases amidst COVID-19 have exacerbated the obstacles faced by the healthcare sector in Kerala, a state with the highest human development index in India (2011) [3,16]. The state which has experienced a shift from public to private healthcare, an increased incidence of non-communicable diseases, and decreased funding with expensive medical treatments is now challenged by a decline in the availability of healthcare infrastructure and resources [3]. Recent climate change and raging floods alongside dense forests have possibly resulted in copious vector-borne infectious diseases [3,14]. Moreover, the migrant crisis has been looming over the state for the past few decades, resulting in the accumulation of various other dangerous diseases from different parts of the world [3,14,16]. Furthermore, symptoms such as fever, malaise, and myalgia are common among COVID-19 patients, and other communicable diseases pose significant challenges in accurate diagnosis and management in resource-limited settings. What is even more concerning, is the high transmission rate of these contagious diseases, thereby placing the frontline healthcare workers at a particularly high risk [9,[13], [14], [15]]. Although the COVID-19 fatality rate in Kerala was as low as 0.51, the test positivity rate was high at 5.58 [6]. The elections in 2020, various festive gatherings which grossly impeded social distancing were likely the reason for the unprecedented burden on the healthcare system [7,16]. The short-lived euphoria of the ideal “Kerala Model” has revealed its flaws which include under-reporting of deaths and low testing capacity in comparison with other Indian states [16]. The ease of restrictions and lack of enforcement of strict regulations may have contributed to the resurgence of both COVID-19 and other infectious diseases [16].

The district of Thrissur reported the third highest number of active COVID-19 cases in the state as of 3 January 2022 with six active cases of the Omicron variant and a recent case of post Kala-azar dermal leishmaniasis (PKDL) in a patient first diagnosed with VL in 2019 [6,11]. Although the first case of visceral leishmaniasis was detected in 2001 in a child from Varantharapilly, Thrissur, multiple other districts also reported cases of the disease [11]. India, which accounts for half of the global burden of VL, identifies it as tricky to eliminate from endemic blocks due to difficult geographical terrain, indigenous populations, poor health-seeking behaviour, and challenges to program implementation. Its symptoms are nonspecific, and suspected cases are identified by ruling out other prevalent febrile conditions, leading to a delay of one month from the incubation period. Consequently, both caseload and mortality remain underestimated owing to poor testing capacity in such areas [17]. The identification of relapsed cases remains confined to a few tertiary health care facilities, exacerbating the challenge of fulfilling the operational definition of elimination as a public health problem.

NiVD, previously detected on 19 May 2018 in Kerala's Kozhikode and Mallapuram districts resulted in 17 deaths and 18 confirmed cases with other similar outbreaks in India during 2001 (66 cases with a 68% mortality rate) and 2007 (5 cases with 100% mortality rate) [10,14]. However, owing to technical shortcomings, poor preparedness, and suboptimal training provided to surveillance personnel, Kerala remains at a relatively high risk for NiVD outbreaks, as identified by the world health organization (WHO) [14]. Despite continual collaborative efforts from the WHO in the past, poor epidemiological surveillance in the state may make it difficult to contain the present spread in 68 NiVD cases [10]. Moreover, Kerala does not have a single laboratory boasting biosafety level 3 (BSL-3) for NiVD testing [14]. Furthermore, lack of treatment or vaccine, as well as neurological sequelae such as seizure disorders that are reported in about 20% of those who survive NiVD-associated encephalitis, present a great strain for the healthcare system [14]. It is noteworthy to highlight the prevalence of fruit bats in South India which are relatively common in Kerala villages and are the natural hosts of the Nipah virus [10,14].

Meanwhile, the state's first Zika virus outbreak identified in the Thiruvananthapuram district has recently declined, although a surge in COVID-19 still poses a health threat [9]. A total of 66 cases of ZVD have been reported, including 13 healthcare workers. Two cases of ZVD were recently reported in Ernakulam, the district which is currently experiencing the highest surge in COVID-19 as of 3rd January 2022 [9]. In the past, ZVD was first reported in Gujarat (2016), besides large outbreaks in Rajasthan and Madhya Pradesh (2018), particularly in pregnant women with post-infection sequelae and complications such as microcephaly of the foetus, intrauterine growth restriction, and even abortion [9].

## 3 Recommendations

Given the gravity and rapid evolution of the endemic plight in South India, it is imperative that policymakers and all relevant stakeholders in the region take immediate action to mitigate further progression of an already obvious situation. Enactment and strict enforcement of preventive measures by local administrative bodies at the grassroots level are some of the most important effective strategies that must be implemented at the earliest.

Government mandated health promotion and awareness campaigns as community outreach programs with the help of educational institutions and organizations may go a long way in helping combat the emerging crisis. Community healthcare workers should help make the local population aware of the importance of proper handwashing techniques, maintenance of hygiene, keeping surroundings clean, social distancing, usage of masks, mosquito nets, and educating the masses about the danger signs and symptoms of these diseases along with aiding in the referral to higher centres for treatment. The focus must primarily lie on effective screening and employing relevant disease control measures for timely and accurate diagnosis to help limit the spread of various diseases. Enforcing standard operating procedures (SOP) which cover aggressive contact tracing, infection control for ambulances, and careful and ideal management of biomedical waste alongside disinfection and sterilisation should also be considered. Close monitoring and epidemiological surveillance using contemporary technology should be emphasised, with immediate cessation of any gathering which may serve as an infectious hotbed for not only COVID-19 but also other highly transmissible diseases. Environmental sampling (such as polluted wastewater from a particular population) as well as clinical samples from infected people, livestock, and wildlife. Regular monitoring of critical reservoirs will help identify peaks in viral concentrations or indicators that, in turn, can be related to early signals of disease outbreaks. Additionally, isolation and quarantine are key formative tools, especially in the context of COVID-19. It is also necessary to constantly follow-up and amplify the screening of rapidly evolving endemic diseases in high-risk groups. Precautionary protective measures, such as providing adequate personal protective equipments (PPE), giving priority to chemoprophylaxis with vaccination of unvaccinated, providing booster dose for the vaccinated with preference to healthcare workers, geriatric population, and those with comorbidities. Adequate sanitary measures and the formulation of laws to protect healthcare workers from violence must be deployed and strengthened to protect frontline personnel. Authorities should also promote research on different diseases and provide financial grants for the development of technologies, including vaccines.

It has been observed that over half of the COVID-19 cases in Kerala can be attributed to ‘super spreaders,’ who constitute only 3% of the population, the prevention of which would drastically decrease transmission, variance in outcome, and resurgence [6]. Equitable vaccine distribution and ‘catch-up’ vaccination should be ensured.

The utilisation of pre-existing government infrastructure, such as the national rural health mission (NRHM) and the national vector borne disease control programme (NVBDCP), must be reinforced [17]. Diagnostic protocols such as parasite screening should also be established and coupled with COVID-19 testing at various venues to differentiate between diseases [18]. Resolute efforts should be geared toward reducing human-animal contact throughout South Indian regions, particularly dense agricultural and rural lands, in order to help curb the spread of vector-borne and zoonotic diseases. In addition to conventional medical treatment, there is a need for a holistic approach, under ‘one-health,’ for the sake of public health and well-being at one with biodiversity and ecosystem conservation, to mitigate emerging infectious diseases.

## 4 Conclusion

With growing evidence of the syndemic nature of COVID-19 with various other infectious diseases, it is of utmost importance to maintain adequate surveillance and monitoring of other infectious diseases, especially in the context of the timeframe wherein an exponential or steep rise in COVID-19 cases can be ascertained. Lastly, strong social, financial, and political commitment toward integrated delivery ‘touch-points’ for public health interventions should be inculcated to ensure timely, well-organized responses to any outbreak.

## Ethical approval

None.

## Source of funding

None.

## Author contribution

All authors have contributed equally to the paper.

## Trail registry number

None.

## Garantor

Utkarsha Uday.

## Declaration of competing interest

None.

## Abbreviations

COVID-19: Coronavirus disease-19
GDP: gross domestic product
ICU: intensive care unit
nCoV: novel Coronavirus
NiVD: Nipah virus disease
NRHM: national rural health mission
NVBDCP: national vector borne disease control programme
PKDL: post Kala-azar dermal leishmaniasis
PPE: personal protective equipment
SARS-CoV-2: severe acute respiratory syndrome Coronavirus-2
SOP: standard operation protocols
VL: visceral leishmaniasis
ZVD: Zika virus disease
